# Gene Expression Profiling Identifies Interferon Signalling Molecules and IGFBP3 in Human Degenerative Annulus Fibrosus

**DOI:** 10.1038/srep15662

**Published:** 2015-10-22

**Authors:** Zepur Kazezian, Rahul Gawri, Lisbet Haglund, Jean Ouellet, Fackson Mwale, Finbarr Tarrant, Peadar O’Gaora, Abhay Pandit, Mauro Alini, Sibylle Grad

**Affiliations:** 1 Centre for Research in Medical Devices (CÚRAM), National University of Ireland, Galway, Ireland; 2 McGill Scoliosis and Spine Group, Department of Surgery, McGill University, Montreal, Canada; 3 Department of Surgery, Lady Davis Institute, McGill University, Montreal, Canada; 4 UCD School of Biomolecular and Biomedical Science, UCD Conway Institute, University College Dublin, Dublin, Ireland; 5 AO Research Institute Davos, Davos, Switzerland; 6 Collaborative Research Partner Annulus Fibrosus Repair Program, AO Foundation, Davos, Switzerland

## Abstract

Low back pain is a major cause of disability especially for people between 20 and 50 years of age. As a costly healthcare problem, it imposes a serious socio-economic burden. Current surgical therapies fail to replace the normal disc in facilitating spinal movements and absorbing load. The focus of regenerative medicine is on identifying biomarkers and signalling pathways to improve our understanding about cascades of disc degeneration and allow for the design of specific therapies. We hypothesized that comparing microarray profiles from degenerative and non-degenerative discs will lead to the identification of dysregulated signalling and pathophysiological targets. Microarray data sets were generated from human annulus fibrosus cells and analysed using IPA ingenuity pathway analysis. Gene expression values were validated by qRT-PCR, and respective proteins were identified by immunohistochemistry. Microarray analysis revealed 238 differentially expressed genes in the degenerative annulus fibrosus. Seventeen of the dysregulated molecular markers showed log_2_-fold changes greater than ±1.5. Various dysregulated cellular functions, including cell proliferation and inflammatory response, were identified. The most significant canonical pathway induced in degenerative annulus fibrosus was found to be the interferon pathway. This study indicates interferon-alpha signalling pathway activation with IFIT3 and IGFBP3 up-regulation, which may affect cellular function in human degenerative disc.

Low back pain (LBP) is a major cause of disability that has become a serious socio-economic burden^1^. Although LBP is common in people who are between 20 and 50 years old, the burden is more persistent in the older population^2^. According to the WHO, LBP is the most costly healthcare problem, and estimates suggest that the total costs exceed $100 billion per year in the United States alone^3^. LBP is commonly linked to the degeneration of the intervertebral disc (IVD)^4^,^5^. Discogenic low back pain is considered to originate from critical tears and fissures in the annulus fibrosus (AF) leading to nerve ingrowth and crucial weakness of the outer annulus that eventually results in a protrusion of nucleus pulposus (NP) tissue^6^. Annular defects may arise from trauma, aging or disc degeneration and may lead to extracellular matrix (ECM) degradation with concomitant loss of proteoglycan, tissue hydration and disc height. This weakens the AF such that it cannot withstand the hydrostatic pressures from the NP nor stabilise the functional spine unit^7^.

Current surgical therapy options include the removal of the degenerated or herniated tissue or even the partial or complete replacement of the disc with an artificial substitute^8^. However, these approaches have considerable drawbacks, such as the risk for adjacent disc degeneration and the failure of the artificial implants to accurately replace the normal disc in terms of movements and absorbing the load pressure^9^,^10^. Therapeutic intervention at an early stage of degeneration could avoid the need for such highly invasive procedures. Although substantial advancement has been achieved in identifying the multifactorial mechanisms that encompass the degenerative flow, knowledge about molecular pathways involved in its initiation and progression is still limited. Therefore, the current focus of regenerative medicine is on identifying biomarkers and signalling pathways that provide a better understanding of the cascades of disc degeneration.

The abnormal cell-mediated response, the changes that occur in the extracellular matrix composition^11^ and the diminished biomechanical characteristics^12^, which can be induced by non-physiological mechanical loading^13^, genetic predisposition and decreased cell activity^14^, lead to gradual structural failure of the IVD. This condition defines the degenerative disc disease (DDD) that goes along with nerve in-growth and low back pain^15^. Disc degeneration is also accompanied by inflammation, which is one of the major factors leading to phenotype changes and apoptosis. During disc degeneration, anabolic metabolism is decreased, whereas the catabolic molecular markers are increased^16^. Importantly, comparison between healthy and degenerated discs shows an imbalance of inflammatory cytokines that significantly increase during the degenerative process. Among the inflammatory cytokines discovered to date in disc degeneration and herniation in patients with severe LBP in comparison with healthy tissues, interleukin (IL)1-beta and tumour necrosis factor (TNF)-alpha are most prominent^17^. During the progression of the degenerative process, many other inflammatory cytokines and catabolic mediators such as prostaglandin E2, nitric oxide, IL6, IL8, matrix metalloproteases (MMPs), a disintegrin and metalloprotease with thrombospondin motif (ADAMTS)4 and ADAMTS5 enzymes and the death-inducing ligand Fas synchronize and consequently degrade the extracellular matrix^18^,^19^.

Recently, the focus has shifted towards identifying signalling pathways affecting the cellular and molecular functions and highlighting the underlying molecular markers for better understanding of the degenerative process in the disc. One of the most important pathways identified recently is the Wnt signalling that may mediate IVD degeneration by activation of MMPs and degradation of matrix molecules leading to NP cell senescence^20^. Moreover, caveolin-1, which regulates the Wnt signalling pathway, was reported to be up-regulated in the human degenerated IVD and was correlated with an increase in cell senescence markers^21^. On the other hand, Smolders *et al.* reported that in the dog NP, caveolin-1 and hence Wnt/β-catenin signalling pathway is crucial for the preservation of notochordal cells and hence for disc regeneration^22^. Furthermore, signalling pathways that regulate pro-inflammatory processes are activated during IVD degeneration and have been reported as potential therapeutic targets. Specifically, nuclear factor kappa B (NF-kB) and mitogen-activated protein kinase (MAPK) pathways have been identified as key regulators of inflammation, matrix catabolism and pain^23^. Recent findings also indicate a role of Notch signalling in the progress of disc degeneration, as Notch receptors and target genes were up-regulated in disc cells following treatment with pro-inflammatory cytokines^24^.

Nevertheless, further investigation into dysregulated processes in degenerative human disc is essential to provide more prospects for therapeutic targets. Large-scale assessment of molecular profiles enables us to comprehensively search for molecular markers and pathways associated with impaired cell functions in the human disc degenerative process. A genome-wide analysis of human AF samples was undertaken by Gruber *et al.* and focussed on the expression of genes associated with pain, neurotrophin and nerve regulation^25^. Significant changes in numerous genes related to these ontologies were found in more degenerated compared to less degenerated discs. In a similar study, microarray analysis was used to identify the expression patterns of genes related to mitochondrial function in human AF specimens, whereby the expression changes indicated mitochondrial dysfunction in degenerative AF^26^. Furthermore, gene expression profiling was utilised to detect differences between notochordal and chondrocyte-like nucleus pulposus cells from non-chondrodystrophic and chondrodystrophic dogs^22^; interestingly, dysregulation of canonical Wnt signalling was associated with early disc degeneration in chondrodystrophic breeds. Recently, several differentially expressed long noncoding RNAs were identified by microarray analysis of degenerative *versus* non-degenerative human NP samples, expanding our understanding of aberrant gene regulation in DDD^27^.

In the present study, a new set of microarray data was generated and analysed from human non-degenerative and degenerative disc cells, with particular emphasis on the annulus fibrosus which is the principal source of discogenic symptoms. Following on from the single gene alterations, we focussed on perturbations in molecular and cellular functions and signalling pathways in the degenerative AF. As such the objectives of this study were (1) to highlight the most dysregulated molecular markers and cellular functions in the human degenerative annulus fibrosus and (2) to identify the activated catabolic pathway(s) through the involved dysregulated molecular markers. The outcomes of this study provide further insight into the cascade of events during disc degeneration in the AF. In particular, results indicate that the interferon-alpha (IFNA) signalling pathway may be involved in mediating degeneration in AF tissue. This pathway may therefore be targeted with a well-designed therapeutic agent at early stages of degeneration.

## Results

### Microarray comparison of degenerative versus non-degenerative human AF

Degeneration of the AF often leads to protrusion and disc herniation. The present work therefore specifically analysed the phenotype changes in degenerative AF cells. The goal is to elucidate AF degenerative pathways to ultimately develop regenerative strategies and improve AF repair. To compare the complete gene expression profiles between cells from degenerative and non-degenerative human AF, Affymetrix® whole genome gene chip microarrays were used. Analysis of the data obtained from the microarray profiling of 16 degenerative and 8 non-degenerative samples led to the identification of 238 significantly differentially expressed genes in the degenerative human annulus fibrosus (Supplementary Table 1). Seventeen of the dysregulated molecular markers showed log_2_-fold change values above the cut off of ±1.5. Out of these 17 molecular markers 10 were up-regulated, while 7 were down-regulated in AF cells of degenerative *versus* non-degenerative discs (Table 1). Insulin-like growth factor binding protein 3 (*IGFBP3*) was identified as the most strongly up-regulated gene in degenerative human AF, indicating a dysregulation of IGF function in degenerative IVD.

### Dysregulated molecular and cellular functions in the degenerative human AF

**Figure 1:**
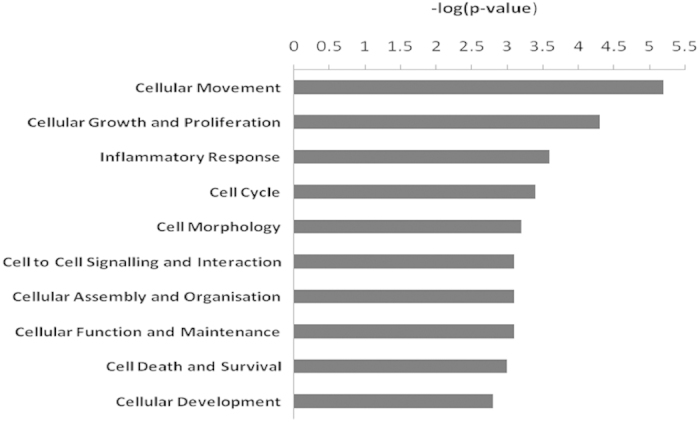
Molecular and cellular functions in the human degenerative *versus* non-degenerative annulus fibrosus. The bar graph image obtained from IPA ingenuity pathway analysis system shows the 10 most dysregulated molecular and cellular functions identified from microarray data of human degenerative *versus* non-degenerative annulus fibrosus. N = 16 for degenerative; N = 8 for non-degenerative samples.

Ingenuity® Pathway Analysis software system (IPA®, QIAGEN Redwood City) was used to identify dysregulations of molecular and cellular functions in degenerative human AF. Analysis of the microarray data by IPA system showed several dysregulated cellular and molecular functions, such as impaired cellular movement, cellular growth and proliferation, inflammatory response, and other disrupted cellular functions (Fig. 1). Out of these main dysregulated cellular functions, the cellular growth and proliferation in correlation with the inflammatory response was analysed in more detail using IPA, as these functions are key determinants of human disc degeneration^16^. The analysis of the genes included in each function/bar graph with the IPA system, showed the interrelation of the inflammatory response via the cytokine interferon-alpha (IFNA) and its signalling pathway, which induces downstream genes such as interferon-induced protein with tetratricopeptide repeats 3 *(IFIT3)*, with the cell growth and proliferation through IFIT3^28^ and IGFBP3; both IFIT3 and IGFBP3 negatively regulate the cell cycle and influence the cell growth and proliferation^29^. This suggests that diminished cell proliferation in degenerative AF might be mediated by interferon induced proteins and loss of IGF function.

### Significant molecules involved in the cell proliferation network of the degenerative human AF

**Figure 2:**
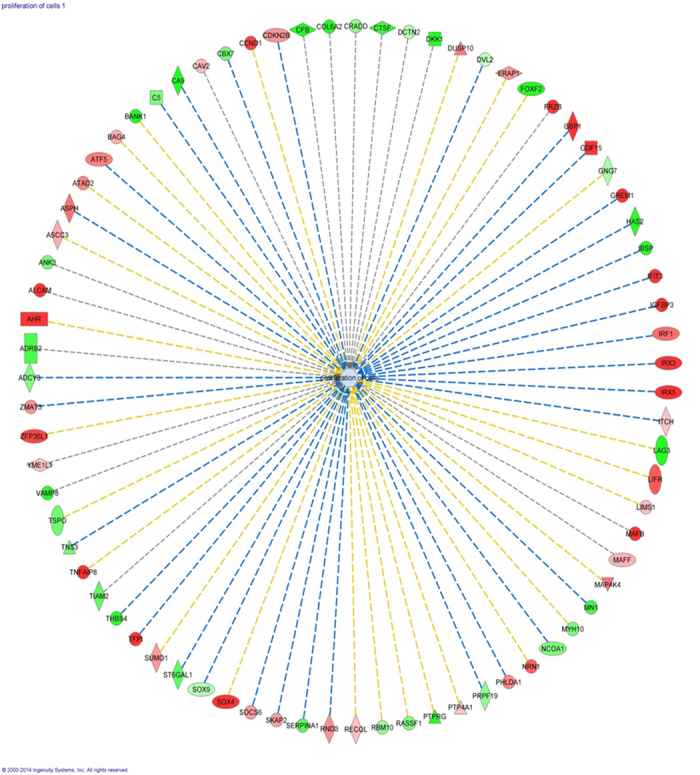
Cell proliferation network in the human degenerative *versus* non-degenerative annulus fibrosus. The scheme is the result of microarray data analysis by the IPA ingenuity pathway analysis system showing 77 significantly dysregulated genes that are involved in the cellular growth and proliferation network. Red represents up-regulation of genes, while green represents down-regulation of genes. Light red or green represent slight up-regulation or down-regulation. Different shapes have different designations as indicated under http://ingenuity.force.com/ipa/articles/Feature_Description/Legend.

**Figure 3:**
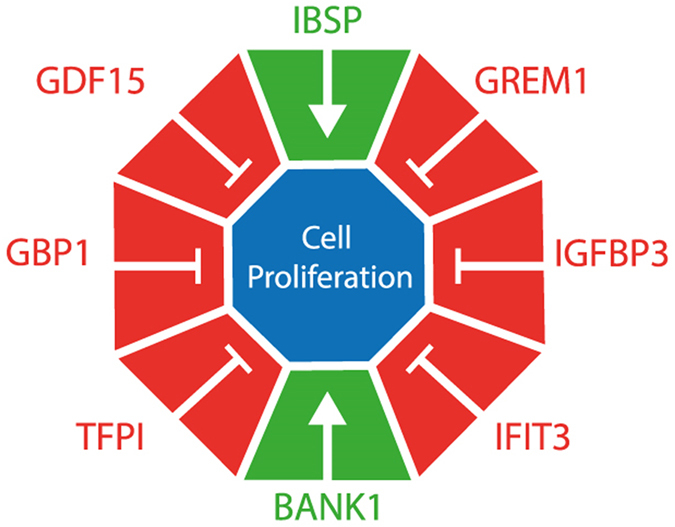
Most dysregulated genes involved in cell proliferation in the human degenerative annulus fibrosus. The eight top dysregulated genes (p < 0.05, log_2_ fold ≥±1.5) were identified within the 77 significantly differently expressed genes (p < 0.05) involved in the cellular growth and proliferation. All the 6 genes up-regulated (red) and the 2 genes down-regulated (green) negatively affect cell proliferation.

Based on the finding of dysregulated cellular growth and proliferation, this function was assessed specifically using the IPA system to comprehensively elucidate proliferation related genes altered in degenerative human AF. Analysis of the microarray data by IPA system revealed 77 significantly differently expressed molecules involved in cellular proliferation (Fig. 2), out of which 8 were listed among the top dysregulated molecular markers (Table 1) and were illustrated in Fig. 3. These data corroborate the role of the differentially expressed molecules in the regulation of cellular growth and proliferation.

### Dysregulated canonical pathways in the degenerative human AF

**Figure 4:**
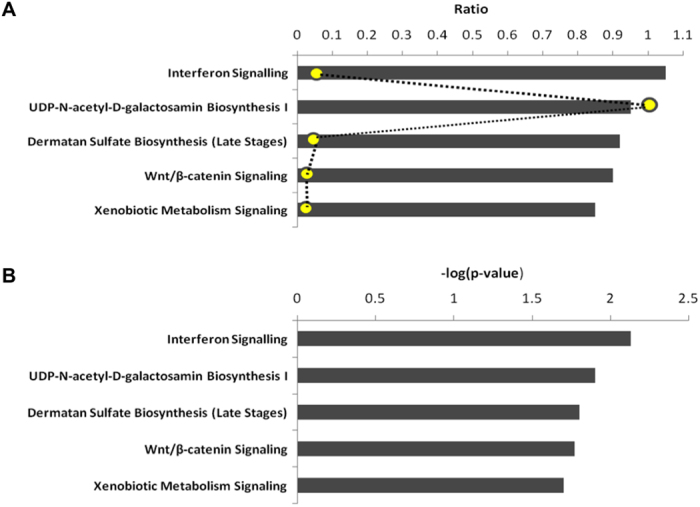
Canonical pathways in the human degenerative annulus fibrosus. The bar graphs in A (scaled according to the ratio) and B (scaled according to the –log (p-value), are representative of the results from IPA ingenuity pathway analysis of microarray data from human degenerative *versus* non degenerative annulus fibrosus, showing the 5 most dysregulated canonical pathways in the degenerative annulus fibrosus. The canonical pathways involve interferon signalling as the top disrupted canonical pathway, followed by UDP-N-acetyl-D-galactosamine biosynthesis 1, dermatan sulfate biosynthesis, Wnt/beta-catenin signalling, and xenobiotic metabolism signalling. The bar graphs are generated according to the number of the significant genes participating in each pathway. The ratios (in yellow) in graph A represent the number of significantly expressed genes over the total number of genes involved in each canonical pathway.

To specifically investigate the signalling pathways that may lead to the up-regulation of *IFIT* genes (Table 1), the molecules involved in the canonical pathways were assessed by IPA. Analysis of the microarray data by IPA system showed several dysregulated canonical pathways (Fig. 4), including interferon (IFN) signalling (3/34), followed by UDP-N-acetyl-D-galactosamine biosynthesis 1 (1/1), dermatan sulfate biosynthesis (3/43), Wnt/beta-catenin signalling (6/174), and xenobiotic metabolism signalling (8/294). Out of these impaired canonical pathways, the inflammatory cytokine interferon mediated signalling pathway was highlighted as the most dysregulated canonical pathway in the degenerated human AF. The results in Fig. 4 were generated by IPA based on the number of significantly differentially expressed genes in relation to the total number of genes included in each pathway. Although the ratio of the significantly dysregulated molecules over the total number of dysregulated molecules involved in the UDP-N-acetyl-D-galactosamine biosynthesis 1 is higher (1/1), the genes involved in the IFN signalling pathway (3/34) are more significant (p = 7.07E-03) than UDP-N-acetyl-D-galactosamine biosynthesis 1 (p = 1.16E-02), ranking the IFN signalling as the top dysregulated pathway. Activation of IFN signalling may therefore play a substantial role in the degenerative human AF.

### RT-PCR analysis of genes dysregulated in degenerative human AF

**Figure 5:**
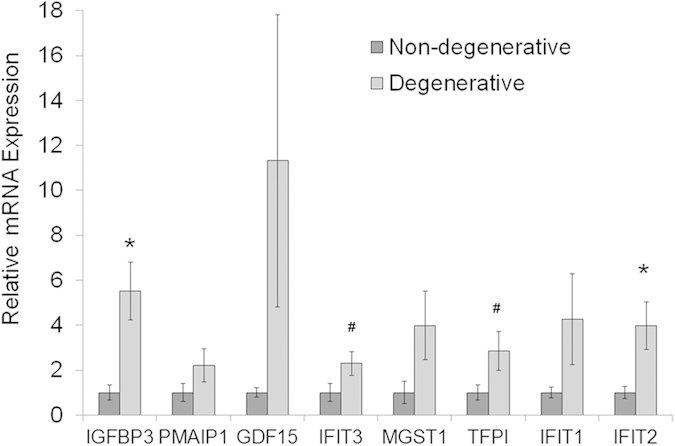
Expression of genes up-regulated in the human degenerative *versus* non-degenerative annulus fibrosus. Real-time RT-PCR data confirm *IGFBP3, IFIT3, TFPI* and *IFIT2* were significantly increased in degenerative annulus fibrosus cells, while *PMAIP1, GDF15, MGST1* and *IFIT1* were up-regulated with no statistical significance. The data represent fold changes ± standard error. For statistical analysis, a two-tailed t-test was carried out on log_2_ transformed relative expression data and multiple testing correction was performed according to the method of Benjamini and Hochberg, with 10% false discovery rate considered significant. N = 10 for degenerative samples; N = 8 for non-degenerative samples; *p < 0.05; #p < 0.1 degenerative *versus* non-degenerative.

Based on the above results and on our interest in the regulation of inflammatory interferon signalling, the difference in expression of selected genes was measured by qRT-PCR analysis. Expression differences were analysed from the most dysregulated genes involved in cellular growth and proliferation and the genes induced by the interferon signalling pathway. As shown in Fig. 5, significant up-regulation in degenerative AF was confirmed for expression levels of *IGFBP3* (5.53 ± 1.29 fold), *IFIT3* (2.29 ± 0.53 fold), tissue factor pathway inhibitor *(TFPI)* (2.85 ± 0.88 fold), and *IFIT2* (3.98 ± 1.05 fold). Furthermore, while mRNA up-regulation was also noted for growth differentiation factor 15 *(GDF15)*, Phorbol-12-myristate-13-acetate-induced protein 1 *(PMAIP1)*, microsomal glutathione S-transferase 1 (MGST1) and *IFIT1*, the difference was not statistically significant (Fig. 5); while no increases were found by qPCR for gremlin 1 *(GREM1)* and guanylate binding protein 1 *(GBP1)* gene expression levels. In addition, the mRNA expression of both integrin-binding sialoprotein *(IBSP)* (ratio degenerative *vs*. non-degenerative 0.062 ± 0.026; p < 0.01) and B-cell scaffold protein with ankyrin repeats 1 *(BANK1)* (0.370 ± 0.070; p < 0.05) were markedly decreased in degenerative AF. These results confirmed the substantial increases in gene expression levels of factors involved in cell proliferation and interferon signalling that were evident from microarray data analyses.

### Immunohistochemistry

**Figure 6:**
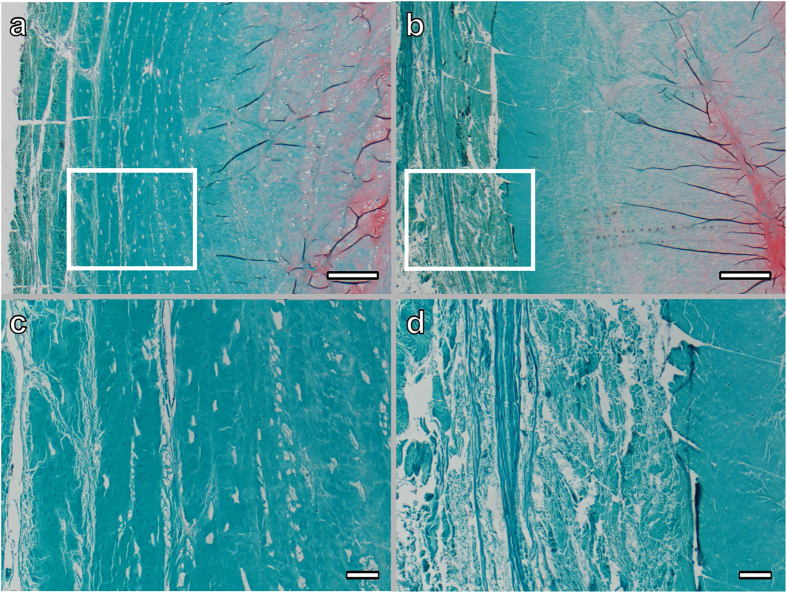
Safranin-O/Fast Green stained sections of annulus fibrosus tissue from a non-degenerative (**a,c**) and a degenerative (**b,d**) disc. Intact fibers with parallel arrangement are noted in the AF of a lumbar disc (degeneration Grade I) from a 13 year old male (**c**); while fibers are loose and arrangement is disrupted in the AF of a lumbar disc from a 47 year old female with disc degeneration Grade III (**d**). Scale bars: 500 μm (**a**,**b**); 100 μm (**c**,**d**).

**Figure 7:**
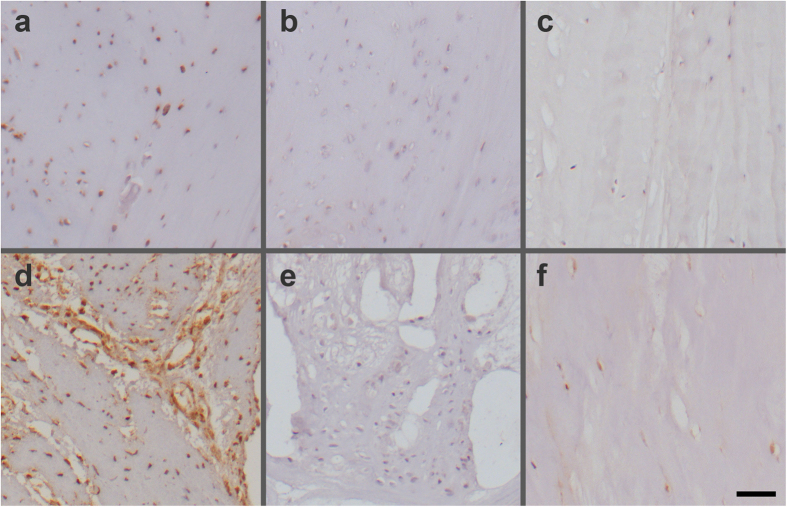
Immunohistochemical analysis of IGFBP3 (**a–c**) and IFIT3 (**d–f**) in sections of human degenerative (**a, b, d, e**) and non-degenerative (**c, f**) annulus fibrosus. Intense immunolabelling for IGFBP3 (**a**) and IFIT3 (**d**) was observed in degenerated regions of the annulus fibrosus of a lumbar disc from a 47 year old female with disc degeneration Grade III. Negative control sections for IGFBP3 (**b**) and IFIT3 (**e**) did not show any staining. IGFBP3 positive cells were absent (**c**), while IFIT3 positive cells were observed in the inner annulus fibrosus of a normal disc (**f**) (degeneration Grade I) from a 13 year old male. Scale bar: 50 μm.

Safranin-O/Fast Green staining was used to qualitatively assess the degeneration of the disc, particularly the annulus fibrosus matrix. In the non-degenerative discs of grade I, collagen fibers in the AF were intact and aligned in parallel, while fibers were disrupted with no clear arrangement in the degenerative discs. Representative sections from discs with reported immunostaining are shown in Fig. 6. IGFBP3 as the most dysregulated molecular marker and IFIT3 as the most dysregulated interferon-induced protein (Table 1) were selected for immunohistochemical detection and localisation of the proteins in human annulus fibrosus. A strong cytoplasmic immunostaining for IGFBP3 and IFIT3 was observed in severely degenerative areas of the human AF (Fig. 7a,d). In areas of only slight degenerative changes, cellular staining for IFIT3 was observed, though a considerable proportion of cells were negative for IFIT3. IGFBP3 immunolabelling was also detectable in cells of less degenerative AF regions, although the staining appeared weak. Furthermore, IGFBP3 positive cells were noted around the vessels occasionally seen in degenerative AF samples. In non-degenerative discs (Grade I), single cells that were positive for IGFBP3 were sporadically detected in the AF, although young AF tissue was mostly negative for IGFBP3 (Fig. 7c); IFIT3 positive cells were also observed in the AF of normal grade I discs (Fig. 7f), although immunostaining was weaker in non-degenerative compared to degenerative discs.

### The interferon signalling pathway activated in the degenerative human AF

**Figure 8:**
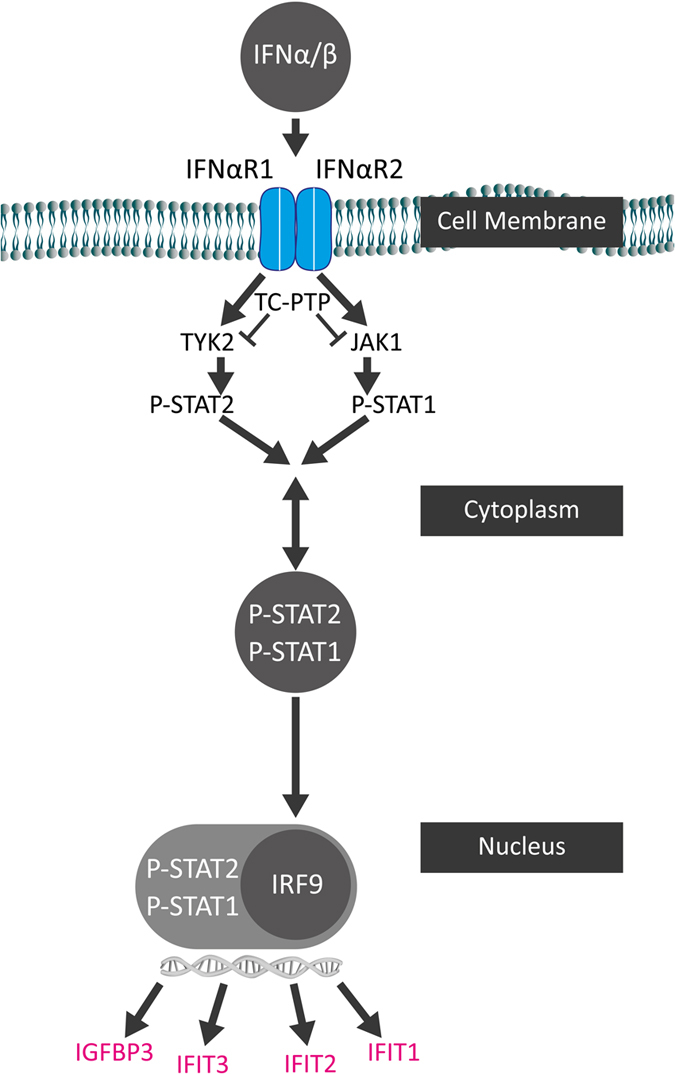
Interferon (IFN) signalling pathway in the human degenerative disc. The schematic generated is representative of the figure produced by IPA ingenuity pathway analysis system that shows the most dysregulated canonical pathway, the IFN signalling pathway, identified by microarray data of human degenerative *versus* non-degenerative annulus fibrosus. Several genes found dysregulated in degenerative annulus fibrosus are induced or up-regulated through IFNA signalling pathway, such as *IFIT1, IFIT2, IFIT3*, as well as *IGFBP3* and are indicated in pink.

The findings throughout the analysis of the current microarray data with IPA ingenuity software were summarized in a common scheme. Combined data indicate that interferon-alpha (IFNA) signalling pathway is activated in the human degenerative annulus fibrosus via induction of 3 IFITs and other genes such as IGFBP3; a respective schematic was adapted from the pathway illustration generated by the IPA system (Fig. 8).

## Discussion

Disc degeneration is a chronic disease that involves different factors leading to the decrease in the number of disc cells and eventually to ECM degradation, neural invasion and low back pain^15^,^30^. To identify the molecular markers that are altered in the degenerative AF due to induced catabolic pathways during disc degeneration, microarray data sets from cells of non-degenerative and degenerative human AF tissues were investigated. To maximise the statistical power of our analysis, we chose to compare non-degenerated samples (grades I-II) to degenerated samples (grades III-V). Individual grade categories lacked sufficient sample numbers to detect significant differences in gene expression. Microarray comparison revealed seventeen molecular markers with log_2_-fold change ≥±1.5, while 238 genes were significantly up-regulated or down-regulated in the human degenerative AF. Moreover, analysis of the major dysregulated functions in the human degenerative AF revealed several dysregulated cellular and molecular mechanisms. Of those dysregulated functions, the cellular growth and proliferation in combination with the inflammatory response were analysed by biostatistical tools to determine the interrelation of these functions. Importantly, out of the main dysregulated molecules in the human AF that are involved in the cell proliferation network, both IGFBP3 and IFIT3 can adversely affect cellular growth and proliferation. Previous work has shown that IFIT3 and IGFBP3 were involved in the regulation of cellular growth and proliferation, exerting anti-proliferative effects on many cell types^31^,^32^,^33^.

In addition to the identification of the molecular markers and the cellular functions, the analysis by IPA system indicates that the IFN signalling pathway was activated, as IFN signalling was highlighted as the main dysregulated canonical pathway. Interferons are among the first cytokines discovered and have widely been investigated in research for interpretation of signalling pathways^34^. Originally, IFNs were identified by their antiviral activities; however, IFNs are nowadays better known for their distinct cellular functions including inhibition of proliferation and angiogenesis, induction of differentiation, and control of the immune system^35^. Because of IFNs’ anti-proliferative effect, they were introduced as a treatment for different medical conditions including viral, tumour, and neurological disorders^36^. The binding of type I IFNs to the IFNA receptors initiates a signalling cascade, which leads to the induction of more than 300 IFN-stimulated genes (ISGs)^37^. Although the presence of the inflammatory cytokine interferon-gamma (IFNG) has been described in herniated disc samples^38^, data about IFNA-related molecules in the context of IVD herniation or degeneration is scarce. According to the microarray data presented in the current study, several IFNA induced genes such as *IFIT1, IFIT2, IFIT3* as well as *IGFBP3* were up-regulated in the AF of the degenerative human discs. This might lead to AF cell growth arrest through the anti-proliferative IFIT3 and to apoptosis via the pro-apoptotic IGFBP3, which negatively regulate the cell cycle and induce apoptosis of the cells directly or indirectly^39^,^40^,^41^,^42^. IGFBP3 is an important regulator of IGF bioavailability that interferes with IGF function and has recently also been associated with the pathogenesis of osteoarthritis^43^. Besides, an IGFBP3 induced indirect effect is to sensitise the cells to cytokine mediated apoptosis signalling including TNFA and IFNG signalling^29^,^33^.

Although high significance was obtained by studying and comparing gene expression in degenerative *versus* non-degenerative human disc tissue, this study is beset with difficulties such as the inability to distinguish between diseased degenerative discs and normal aged discs, by the lack of identical grouping of the discs according to the age of the donors and the level of degeneration, and by the lack of absolute differentiation between symptomatic and asymptomatic degenerated discs. Moreover, although previous studies in rat, canine and human species did not reveal major differences in disc marker gene expression patterns between RNA extracted from isolated cells and RNA extracted directly from the tissues^44^,^45^,^46^,^47^, enzymatic cell isolation might have reduced or masked some degeneration related expression changes of certain genes.

In conclusion, this study indicates that among various degenerative processes in the disc, the IFN signalling pathway is a primarily dysregulated and significantly activated pathway in the degenerative human annulus fibrosus. Up-regulations of IFNA-induced IFITs as well as IGFBP3 are likely to negatively regulate the cell cycle, and hence decrease the disc cell number, eventually accelerating degeneration. Further studies are needed to investigate the role of IFNA signalling in disc degeneration through *in vitro* and *ex vivo* analysis of IFNA effects on disc cells and organ cultures and dissection of the signalling pathway by gene and protein expression analysis.

## Methods

### Collection and processing of human disc tissue samples

Intervertebral disc tissue was obtained by McGill Scoliosis and Spine Group from human lumbar discs through organ donation program of Transplant Quebec in accordance with the local and institutional ethical guidelines. The study was approved by McGill University Institutional Review Board (IRB# A04-M53-08B). Consent was obtained from family members of the donors. The disc tissue was harvested from spinal segments from levels T12-L1 to L4-L5; NP and AF regions were carefully dissected and separated. AF samples were collected from 6 male and 6 female donors, aged between 21 and 82 years (Table 2; samples ID 1–18) within a maximum of 12 hours after declaring brain death.

X-rays were taken from the harvested spines, and the degree of degeneration was evaluated macroscopically by dissecting the discs from the vertebral bodies and examining for features such as loss of regional demarcation and fissures according to the Thompson grading^48^. The grading was done by two independent observers; based on the grading, the discs from 5 donors were then assigned to the control/non-degenerative group having degeneration grades I–II, whereas the discs from 7 donors were assigned to the degenerative group, having grades III–V. A cell isolation step was included before RNA extraction in order to obtain RNA of sufficient quality and quantity suitable for microarray profiling. Cells were isolated from the tissue using sequential Pronase (Roche) and type II collagenase (Worthington Biochemical) digestion. Briefly, AF was minced, treated with 0.2% Pronase for 1 hour, and then with 0.04% collagenase for 6 hours. After enzymatic isolation, cell suspensions were filtered through a 100 μm cell strainer, washed twice with Dulbecco’s modified Eagle’s medium (DMEM), and lysed in Trizol Reagent® (Life Technologies). Total RNA was isolated using a chloroform extraction method followed by purification through the SV Total RNA Isolation System (Promega).

### Microarray profiling and data analysis

Nanodrop (Thermo Scientific) and Bioanalyzer (Agilent) measurements were used to assess the quantity and quality of the RNA samples (Table 2). Samples were processed and profiled using the Human GeneChip U133 Plus 2.0 Affymetrix® arrays as described in previous work^47^. For statistical comparison of degenerative (grade III-V) versus non-degenerative (grade I-II) samples, data generated during an earlier study were included (Table 2; donor ID 13–18; samples ID 19-24)^47^, resulting in a total of 16 samples for the degenerative group and 8 samples for the non-degenerative control group. The statistical environment R (http://www.r-project.org) for Bioconductor (http://www.bioconductor.org)^49^ and the MBNI probe set remappings^50^ were used for data analysis. Samples were background corrected, log2- transformed, and quantile-normalized using the gcrma^51^ and affy^52^ Bioconductor packages. To overcome the batch effect, a Bayesian adjustment of the expression matrix was carried out using the ComBat approach as implemented in the sva Bioconductor package for the R statistical environment. Prior to batch effect removal, the dataset was filtered to remove probe sets not expressed or expressed at very low levels in greater than 80% of samples. Expression differences between the groups were detected using the limma^53^ package with multiple testing correction performed according to the method of Benjamini and Hochberg^54^. Genes with adjusted p < 0.05 were considered significant. Gene array data were uploaded on the Gene Expression Omnibus (GEO) database and are accessible under GSE70362.

Microarray data were further analysed through the use of QIAGEN’s Ingenuity® Pathway Analysis software (IPA®, QIAGEN Redwood City, www.qiagen.com/ingenuity). The input data, composed of 238 significantly differently expressed genes with their log_2_ fold change and p-values (Supplementary Table 1), were uploaded into IPA system for the purpose of mapping the genes onto networks, functions and pathways. The Ingenuity Pathway Analysis (IPA) is based on sample phenotype as well as on disease, cellular, molecular and sequence mechanisms. The analysis utilises specific tools to evaluate the data to produce networks and pathways based on molecular and cellular mechanisms after assigning certain cut offs for significance values.

### Gene expression by qRT-PCR

Significantly dysregulated genes with at least ±1.5 log_2_ fold difference between non-degenerative and degenerative AF after microarray analysis were further quantified by real-time RT-PCR. mRNA samples from human AF cells of n = 8 non-degenerative and n = 10 degenerative discs (Table 2, column qRT-PCR) were used for complementary DNA synthesis and processed by TaqMan Gene Expression Assays (Applied Biosystems), using the following primer/probe systems: IGFBP3-Hs00365742_g1, GREM1-Hs01879841_s1, PMAIP1-Hs00560402_m1, GDF15-Hs00171132_m1, IFIT3-Hs01922752_s1, MGST1-Hs00220393_m1, TFPI-Hs00196731_m1, IFIT1-Hs01911452_s1, IFIT2-Hs01922738_s1, GBP1-Hs00977005_m1, IBSP-Hs00173720_m1, BANK1-Hs01009378_m1. PCR was performed using TaqMan® Gene Expression Master Mix (Applied Biosystems) and standard thermal conditions (10 minutes at 95 °C for polymerase activation, followed by 40 cycles of 95 °C for 15 seconds and 60 °C for 60 seconds). Expression of target genes was normalized to the average of the 3 endogenous controls glyceraldehyde phosphate dehydrogenase (GAPDH-Hs99999905_m1), hypoxanthine phosphoribosyltransferase 1 (HPRT1-Hs99999909_m1) and glucuronidase beta (GUSB-Hs99999908_m1). Gene expression was calculated according to the ∆Ct method^55^. For the statistical analysis, relative expression data were log_2_ transformed, and multiple testing correction was performed according to the method of Benjamini and Hochberg^54^.

### Histology and immunohistochemistry

Human intervertebral discs were collected and scored as outlined above (Table 2). Whole human discs were isolated as previously described^56^ retaining cartilaginous endplates. The discs were then fixed in 70% methanol for 5 days at 4 °C, sealed in dialysis bags (Spectr/Por 3, 3500 kDa molecular weight cut-off dialysis membrane) and decalcified with 12.5% neutral ethylenediaminetetraacetic acid (EDTA) at 4 °C for 21 days with EDTA solution changed every third day. The decalcified discs were then dehydrated in an ascending ethanol gradient, and embedded in paraffin; sections of 6 μm were cut by microtome. Sections were deparaffinised and stained with 0.1% Safranin-O and 0.02% Fast Green to assess proteoglycan and collagen distribution.

For immunohistochemistry deparaffinised sections were first treated with 0.3% hydrogen peroxide in methanol for 30 min. Antigen retrieval was performed by heating the sections (95 °C) in citrate buffer (pH 6.0) for 15 min. Then sections were blocked with 5% normal goat serum for 1 h and treated with anti-IGFBP3 antibody (Acris Antibodies, cat. nr. AP14347PU-N; 6.25 μg/mL) or anti-IFIT3 antibody (Aviva Systems, cat. nr. ARP46034_P050; 5 μg/mL) for 30 min at room temperature. Negative control sections were incubated with rabbit IgG isotype control (Vector Laboratories, Burlingame, USA) according to the manufacturer’s protocol. Biotinylated secondary antibody (Vectastain ABC Elite, Vector Laboratories) was applied at 1:200 dilution, followed by ABC complex, and chromogen was developed using diaminobenzidine (ImmPACT DAB, Vector Laboratories). Counterstaining was carried out using Mayer’s haematoxylin.

## Additional Information

**How to cite this article**: Kazezian, Z. *et al.* Gene Expression Profiling Identifies Interferon Signalling Molecules and IGFBP3 in Human Degenerative Annulus Fibrosus. *Sci. Rep.* **5**, 15662; doi: 10.1038/srep15662 (2015).

## Supplementary Material

Supplementary Information

## Figures and Tables

**Table 1:** Microarray gene expression comparison of human annulus fibrosus cells.

**Symbol**	**Description**	**p-value**	**Log2 fold**
*IGFBP3*	Insulin-like growth factor binding protein 3	0.003	2.85
*GREM1*	Gremlin 1	0.002	2.54
*PMAIP1*	Phorbol-12-myristate-13-acetate-induced protein 1	0.010	2.49
*GDF15*	Growth differentiation factor 15	0.003	2.29
*IFIT3*	Interferon-induced protein with tetratricopeptide repeats 3	0.004	2.03
*MGST1*	Microsomal glutathione S-transferase 1	0.001	2.02
*TFPI*	Tissue factor pathway inhibitor	0.002	2.01
*IFIT1*	Interferon-induced protein with tetratricopeptide repeats 1	0.013	1.96
*IFIT2*	Interferon-induced protein with tetratricopeptide repeats 2	0.032	1.89
*GBP1*	Guanylate binding protein 1, interferon inducible	0.006	1.86
*GPR64*	G protein-coupled receptor 64	0.022	−1.59
*SOD3*	Superoxide dismutase 3, extracellular	0.002	−1.64
*CHAD*	Chondroadherin	0.020	−1.80
*LOXL4*	Lysyl oxidase-like 4	0.032	−1.83
*LYVE1*	Lymphatic vessel endothelial hyaluronan receptor 1	0.011	−1.85
*BANK1*	B-cell scaffold protein with ankyrin repeats 1	0.006	−2.09
*IBSP*	Integrin-binding sialoprotein	0.002	−3.46

Significantly differentially expressed genes with p < 0.05, log2 fold cut off ≥±1.5 (degenerative (n = 16) *versus* non-d*e*generative (n = 8)).

**Table 2:** Details of annulus fibrosus cell donors and corresponding RNA samples used for microarray profiling and qRT-PCR analysis (samples 1–27); details of whole disc donors and corresponding samples used for immunohistochemistry (samples 28–36).

**Sample ID**	**Donor ID**	**Gender**	**Age (y)**	**DD Grade**	**RNA_abs 260_280**	**RNA_abs 260_230**	**RIN**	**QRT-PCR**
1	1	M	73	III	2.12	1.99	7.8	x
2	1	M	73	IV	2.14	1.91	7.9	x
3	2	M	79	III	2.14	1.31	7.6	x
4	2	M	79	IV	2.14	1.68	7.8	
5	3	M	67	IV	2.17	1.85	8.0	x
6	3	M	67	V	2.19	2.04	7.9	x
7	4	F	76	IV	2.18	2.02	7.6	x
8	4	F	76	V	2.22	0.83	7.8	
9	5	F	81	III	2.15	1.87	8.4	x
10	5	F	81	IV	2.18	1.22	8.4	x
11	6	F	44	I	2.19	2.05	6.9	x
12	6	F	44	II	2.17	1.57	6.6	x
13	7	M	75	V	2.17	1.08	8.0	x
14	8	F	40	II	2.17	2.10	7.9	x
15	9	M	82	V	2.15	1.99	4.3	x
16	10	M	24	I	2.13	2.10	8.2	
17	11	F	23	I–II	2.14	2.73	8.5	x
18	12	F	21	I	2.12	0.84	8.5	x
19	13	F	61	III	2.16	2.05	9.2	
20	14	M	72	IV	2.14	2.12	8.7	
21	15	F	81	III	2.11	2.11	9.1	
22	16	M	25	I	2.11	2.08	8.1	
23	17	M	56	III	2.13	1.77	7.8	
24	18	M	32	I–II	2.14	2.05	8.5	
25	19	F	20	I	2.16	1.73	nd	x
26	20	F	16	I	2.2	1.55	nd	x
27	21	F	20	I	2.17	2.05	nd	x
Level								
28	22	M	25	II	T12-L1			
29	23	F	47	III	L2-L3			
30	24	F	42	II–III	L4-L5			
31	25	F	10	I	L4-L5			
32	26	F	34	II	L5-S1			
33	27	M	13	I	T11-T12			
34	27	M	13	I	T12-L1			
35	27	M	13	I	L4-L5			
36	28	F	47	III–IV	T12-L1			
