# Supplementary material for: Gene Expression Profiling Identifies Interferon Signalling Molecules and IGFBP3 in Human Degenerative Annulus Fibrosus
Source: Sci Rep. 2015 Oct 22;5:15662. doi: 10.1038/srep15662 (PMC4614807; doi:10.1038/srep15662)
Supplement: Supplementary Information [file srep15662-s1.doc]

**Gene Expression Profiling Identifies Interferon Signalling Molecules and IGFBP3 in Human Degenerative Annulus Fibrosus**

Zepur Kazezian^1,6^, Rahul Gawri^2,3^, Lisbet Haglund^2^, Jean Ouellet^2^, Fackson Mwale^3^, Finbarr Tarrant^4^, Peadar O'Gaora^4^, Abhay Pandit^1,6^, Mauro Alini^5,6^, Sibylle Grad^5,6*^

^1^ Centre for Research in Medical Devices (CURAM), National University of Ireland, Galway, Ireland

^2^ McGill Scoliosis and Spine Group, Department of Surgery, McGill University, Montreal, Canada

^3^ Department of Surgery, Lady Davis Institute, McGill University, Montreal, Canada

^4^ UCD School of Biomolecular and Biomedical Science, UCD Conway Institute, University College Dublin, Dublin, Ireland

^5^ AO Research Institute Davos, Davos, Switzerland

^6^ Collaborative Research Partner Annulus Fibrosus Repair Program, AO Foundation, Davos, Switzerland

*Address of correspondence:

Sibylle Grad, PhD

AO Research Institute Davos

Clavadelerstrasse 8

7270 Davos

Switzerland

Phone: +41 81 414 24 80

Fax: +41 81 414 22 88

sibylle.grad@aofoundation.org

**Supplementary table 1.** Microarray gene expression comparison of human annulus fibrosus cells. Significantly differently expressed genes with p<0.05 from microarray data. (degenerative (n=16) *versus* non-degenerative (n=8)).

| **Probe** | **Symbol** | **Description** | **log_2_ fold Change** | **p-value** |
| --- | --- | --- | --- | --- |
| 26230_at | *TIAM2* | T-cell lymphoma invasion and metastasis 2 | -0.86 | 5.86E-04 |
| 196_at | *AHR* | Aryl hydrocarbon receptor | 1.4 | 5.86E-04 |
| 29094_at | *LGALSL* | Lectin, galactoside-binding-like | -1.04 | 6.65E-04 |
| 4257_at | *MGST1* | Microsomal glutathione S-transferase 1 | 2.02 | 0.001 |
| 9448_at | *MAP4K4* | Mitogen-activated protein kinase kinase kinase kinase 4 | 0.91 | 0.002 |
| 2295_at | *FOXF2* | Forkhead box F2 | -1.38 | 0.002 |
| 29028_at | *ATAD2* | ATPase family, AAA domain containing 2 | 0.77 | 0.002 |
| 6649_at | *SOD3* | Superoxide dismutase 3, extracellular | -1.64 | 0.002 |
| 3381_at | *IBSP* | Integrin-binding sialoprotein | -3.46 | 0.002 |
| 29941_at | *PKN3* | Protein kinase N3 | -1.45 | 0.002 |
| 7035_at | *TFPI* | Tissue factor pathway inhibitor (lipoprotein-associated coagulation inhibitor) | 2.01 | 0.002 |
| 26585_at | *GREM1* | Gremlin 1 | 2.54 | 0.002 |
| 9530_at | *BAG4* | BCL2-associated athanogene 4 | 0.53 | 0.002 |
| 3486_at | *IGFBP3* | Insulin-like growth factor binding protein 3 | 2.85 | 0.003 |
| 9518_at | *GDF15* | Growth differentiation factor 15 | 2.29 | 0.003 |
| 51114_at | *ZDHHC9* | Zinc finger, DHHC-type containing 9 | -0.47 | 0.003 |
| 6480_at | *ST6GAL1* | ST6 beta-galactosamide alpha-2,6-sialyltranferase 1 | -0.88 | 0.003 |
| 64221_at | *ROBO3* | Roundabout, axon guidance receptor, homolog 3 (Drosophila) | -0.96 | 0.003 |
| 257240_at | *KLHL34* | Kelch-like 34 (Drosophila) | -1.08 | 0.004 |
| 23070_at | *FTSJD2* | FtsJ methyltransferase domain containing 2 | -0.6 | 0.004 |
| 3437_at | *IFIT3* | Interferon-induced protein with tetratricopeptide repeats 3 | 2.03 | 0.004 |
| 374378_at | *GALNTL4* | UDP-N-acetyl-alpha-D-galactosamine:polypeptide N-acetylgalactosaminyltransferase-like 4 | -0.97 | 0.004 |
| 10730_at | *YME1L1* | YME1-like 1 (S. cerevisiae) | 0.36 | 0.004 |
| 55435_at | *AP1AR* | Adaptor-related protein complex 1 associated regulatory protein | 0.97 | 0.005 |
| 139065_at | *SLITRK4* | SLIT and NTRK-like family, member 4 | 1.34 | 0.005 |
| 114294_at | *LACTB* | Lactamase, beta | 0.83 | 0.005 |
| 5911_at | *RAP2A* | RAP2A, member of RAS oncogene family | 0.83 | 0.005 |
| 5793_at | *PTPRG* | Protein tyrosine phosphatase, receptor type, G | -1.28 | 0.005 |
| 10654_at | *PMVK* | Phosphomevalonate kinase | -0.82 | 0.005 |
| 51752_at | *ERAP1* | Endoplasmic reticulum aminopeptidase 1 | 0.68 | 0.005 |
| 10106_at | *CTDSP2* | CTD (carboxy-terminal domain, RNA polymerase II, polypeptide A) small Phosphatase 2 | -0.88 | 0.005 |
| 9403_at | *Sep-15* | 15 kDa selenoprotein | 0.44 | 0.005 |
| 439921_at | *MXRA7* | Matrix-remodelling associated 7 | -1.05 | 0.005 |
| 2633_at | *GBP1* | Guanylate binding protein 1, interferon-inducible | 1.86 | 0.006 |
| 55024_at | *BANK1* | B-cell scaffold protein with ankyrin repeats 1 | -2.09 | 0.006 |
| 10265_at | *IRX5* | Iroquois homeobox 5 | 1.36 | 0.006 |
| 3977_at | *LIFR* | Leukemia inhibitory factor receptor alpha | 1.06 | 0.006 |
| 444_at | *ASPH* | Aspartate beta-hydroxylase | 0.9 | 0.007 |
| 79624_at | *C6orf211* | Chromosome 6 open reading frame 211 | 0.59 | 0.007 |
| 3902_at | *LAG3* | Lymphocyte-activation gene 3 | -1.23 | 0.007 |
| 9517_at | *SPTLC2* | Serine palmitoyltransferase, long chain base subunit 2 | -0.73 | 0.007 |
| 23324_at | *MAN2B2* | Mannosidase, alpha, class 2B, member 2 | -0.88 | 0.007 |
| 9306_at | *SOCS6* | Suppressor of cytokine signaling 6 | 0.63 | 0.007 |
| 27286_at | *SRPX2* | Sushi-repeat containing protein, X-linked 2 | -1.29 | 0.007 |
| 114908_at | *TMEM123* | Transmembrane protein 123 | 0.74 | 0.007 |
| 10973_at | *ASCC3* | Activating signal cointegrator 1 complex subunit 3 | 0.53 | 0.008 |
| 64393_at | *ZMAT3* | Zinc finger, matrin-type 3 | 0.69 | 0.008 |
| 7060_at | *THBS4* | Thrombospondin 4 | -1.16 | 0.008 |
| 10171_at | *RCL1* | RNA terminal phosphate cyclase-like 1 | -0.78 | 0.008 |
| 30837_at | *SOCS7* | Suppressor of cytokine signaling 7 | -0.36 | 0.008 |
| 26127_at | *FGFR1OP2* | FGFR1 oncogene partner 2 | 0.5 | 0.009 |
| 57761_at | *TRIB3* | Tribbles homolog 3 (Drosophila) | 1.14 | 0.009 |
| 2664_at | *GDI1* | GDP dissociation inhibitor 1 | -0.51 | 0.01 |
| 5366_at | *PMAIP1* | phorbol-12-myristate-13-acetate-induced protein 1 | 2.49 | 0.01 |
| 8722_at | *CTSF* | Cathepsin F | -1.06 | 0.011 |
| 10894_at | *LYVE1* | Lymphatic vessel endothelial hyaluronan receptor 1 | -1.85 | 0.011 |
| 51131_at | *PHF11* | PHD finger protein 11 | -0.67 | 0.011 |
| 79959_at | *CEP76* | Centrosomal protein 76kDa | 0.76 | 0.011 |
| 79609_at | *METTL21D* | Methyltransferase like 21D | 0.65 | 0.011 |
| 10440_at | *TIMM17A* | Translocase of inner mitochondrial membrane 17 homolog A (yeast) | 0.62 | 0.011 |
| 8534_at | *CHST1* | Carbohydrate (keratan sulfate Gal-6) sulfotransferase 1 | -1.03 | 0.011 |
| 8673_at | *VAMP8* | Vesicle-associated membrane protein 8 (endobrevin) | -1.23 | 0.011 |
| 1292_at | *COL6A2* | collagen, type VI, alpha 2 | -1.22 | 0.011 |
| 548645_at | *DNAJC25* | DnaJ (Hsp40) homolog, subfamily C , member 25 | 0.86 | 0.012 |
| 10123_at | *ARL4C* | ADP-ribosylation factor-like 4C | 1.39 | 0.012 |
| 51569_at | *UFM1* | Ubiquitin-fold modifier 1 | 0.6 | 0.013 |
| 23186_at | *RCOR1* | REST corepressor 1 | 0.48 | 0.013 |
| 3659_at | *IRF1* | Interferon regulatory factor 1 | 0.94 | 0.013 |
| 3434_at | *IFIT1* | Interferon-induced protein with tetratricopeptide repeats 1 | 1.96 | 0.013 |
| 23492_at | *CBX7* | Chromobox homolog 7 | -0.74 | 0.013 |
| 8935_at | *SKAP2* | Src kinase associated phosphoprotein 2 | 0.62 | 0.013 |
| 2582_at | *GALE* | UDP-galactose-4-epimerase | -0.74 | 0.013 |
| 858_at | *CAV2* | Caveolin 2 | 0.46 | 0.013 |
| 9902_at | *MRC2* | Mannose receptor, C type 2 | -0.91 | 0.014 |
| 706_at | *TSPO* | Translocator protein (18kDa) | -0.77 | 0.014 |
| 595_at | *CCND1* | Cyclin D1 | 1.22 | 0.014 |
| 6322_at | *SCML1* | Sex comb on midleg-like 1 (Drosophila) | 0.44 | 0.014 |
| 9371_at | *KIF3B* | Kinesin family member 3B | -0.4 | 0.015 |
| 27339_at | *PRPF19* | PRP19/PSO4 pre-mRNA processing factor 19 homolog (S. cerevisiae) | -0.6 | 0.016 |
| 5998_at | *RGS3* | Regulator of G-protein signaling 3 | -0.68 | 0.016 |
| 582_at | *BBS1* | Bardet-Biedl syndrome 1 | -0.4 | 0.016 |
| 57683_at | *ZDBF2* | Zinc finger, DBF-type containing 2 | 0.93 | 0.016 |
| 4595_at | *MUTYH* | MutY homolog (E. coli) | -0.55 | 0.017 |
| 3987_at | *LIMS1* | LIM and senescent cell antigen-like domains 1 | 0.41 | 0.018 |
| 51309_at | *ARMCX1* | Armadillo repeat containing, X-linked 1 | 0.5 | 0.018 |
| 288_at | *ANK3* | Ankyrin 3, node of Ranvier (ankyrin G) | -0.74 | 0.018 |
| 340719_at | *NANOS1* | Nanos homolog 1 (Drosophila) | 1.45 | 0.019 |
| 283208_at | *P4HA3* | Prolyl 4-hydroxylase, alpha polypeptide III | -1.13 | 0.019 |
| 55623_at | *THUMPD1* | THUMP domain containing 1 | 0.44 | 0.019 |
| 629_at | *CFB* | Complement factor B | -1.24 | 0.019 |
| 642273_at | *FAM110C* | Family with sequence similarity 110, member C | 1.16 | 0.02 |
| 114785_at | *MBD6* | Methyl-CpG binding domain protein 6 | -0.39 | 0.02 |
| 203069_at | *R3HCC1* | R3H domain and coiled-coil containing 1 | -0.57 | 0.02 |
| 8648_at | *NCOA1* | Nuclear receptor coactivator 1 | -0.68 | 0.02 |
| 284361_at | *EMC10* | ER membrane protein complex subunit 10 | -0.7 | 0.02 |
| 9532_at | *BAG2* | BCL2-associated athanogene 2 | 0.82 | 0.02 |
| 1101_at | *CHAD* | Chondroadherin | -1.8 | 0.02 |
| 738_at | *VPS51* | Vacuolar protein sorting 51 homolog (S. cerevisiae) | -0.73 | 0.02 |
| 7738_at | *ZNF184* | zinc finger protein 184 | 0.78 | 0.02 |
| 4023_at | *LPL* | lipoprotein lipase | -1.32 | 0.02 |
| 4628_at | *MYH10* | myosin, heavy chain 10, non-muscle | -0.71 | 0.02 |
| 6646_at | *SOAT1* | sterol O-acyltransferase 1 | 0.93 | 0.02 |
| 643911_at | *CRNDE* | colorectal neoplasia differentially expressed (non-protein coding) | 0.46 | 0.02 |
| 643641_at | *ZNF862* | Zinc finger protein 862 | -0.45 | 0.02 |
| 89891_at | *WDR34* | WD repeat domain 34 | -0.8 | 0.02 |
| 4189_at | *DNAJB9* | DnaJ (Hsp40) homolog, subfamily B, member 9 | 0.75 | 0.02 |
| 435_at | *ASL* | Argininosuccinate lyase | -0.88 | 0.02 |
| 2788_at | *GNG7* | Guanine nucleotide binding protein (G protein), gamma 7 | -0.45 | 0.02 |
| 9486_at | *CHST10* | Carbohydrate sulfotransferase 10 | -1.29 | 0.021 |
| 2487_at | *FRZB* | Frizzled-related protein | 1.32 | 0.021 |
| 26097_at | *CHTOP* | Chromatin target of PRMT1 | -0.32 | 0.021 |
| 26225_at | *ARL5A* | ADP-ribosylation factor-like 5A | 0.46 | 0.022 |
| 81035_at | *COLEC12* | Collectin sub-family member 12 | -1.19 | 0.022 |
| 10149_at | *GPR64* | G protein-coupled receptor 64 | -1.59 | 0.022 |
| 203_at | *AK1* | Adenylate kinase 1 | -0.57 | 0.022 |
| 10286_at | *BCAS2* | Breast carcinoma amplified sequence 2 | 0.43 | 0.022 |
| 8241_at | *RBM10* | RNA binding motif protein 10 | -0.58 | 0.022 |
| 221178_at | *SPATA13* | Spermatogenesis associated 13 | -0.55 | 0.022 |
| 55556_at | *ENOSF1* | Enolase superfamily member 1 | -0.74 | 0.022 |
| 360023_at | *ZBTB41* | Zinc finger and BTB domain containing 41 | 0.81 | 0.022 |
| 5446_at | *PON3* | Paraoxonase 3 | -1.14 | 0.022 |
| 56180_at | *MOSPD1* | Motile sperm domain containing 1 | 0.55 | 0.022 |
| 54994_at | *GID8* | GID complex subunit 8 homolog (S. cerevisiae) | -0.44 | 0.023 |
| 28513_at | *CDH19* | Cadherin 19, type 2 | 0.74 | 0.023 |
| 3037_at | *HAS2* | Hyaluronan synthase 2 | -1.15 | 0.023 |
| 130814_at | *PQLC3* | PQ loop repeat containing 3 | -0.97 | 0.024 |
| 124936_at | *CYB5D2* | Cytochrome b5 domain containing 2 | -0.65 | 0.024 |
| 282809_at | *POC1B* | POC1 centriolar protein homolog B (Chlamydomonas) | 0.77 | 0.024 |
| 79667_at | *FLJ13197* | Uncharacterized FLJ13197 | -1.44 | 0.024 |
| 205327_at | *C2orf69* | Chromosome 2 open reading frame 69 | 0.43 | 0.024 |
| 29952_at | *DPP7* | Dipeptidyl-peptidase 7 | -0.58 | 0.024 |
| 80264_at | *ZNF430* | Zinc finger protein 430 | 0.73 | 0.024 |
| 22928_at | *SEPHS2* | Selenophosphate synthetase 2 | 0.67 | 0.024 |
| 10656_at | *KHDRBS3* | KH domain containing, RNA binding, signal transduction associated 3 | 0.89 | 0.025 |
| 55070_at | *DET1* | De-etiolated homolog 1 (Arabidopsis) | -0.81 | 0.025 |
| 26258_at | *BLOC1S6* | Biogenesis of lysosomal organelles complex-1, subunit 6, pallidin | 0.44 | 0.026 |
| 22809_at | *ATF5* | Activating transcription factor 5 | 0.82 | 0.026 |
| 85027_at | *C5orf62* | Chromosome 5 open reading frame 62 | 1.53 | 0.026 |
| 214_at | *ALCAM* | Activated leukocyte cell adhesion molecule | 1.31 | 0.026 |
| 5965_at | *RECQL* | RecQ protein-like (DNA helicase Q1-like) | 0.41 | 0.028 |
| 3916_at | *LAMP1* | Lysosomal-associated membrane protein 1 | -0.25 | 0.028 |
| 7321_at | *UBE2D1* | Ubiquitin-conjugating enzyme E2D 1 | 0.75 | 0.028 |
| 51065_at | *RPS27L* | Ribosomal protein S27-like | 0.7 | 0.028 |
| 339324_at | *ZNF260* | Zinc finger protein 260 | 0.74 | 0.028 |
| 26275_at | *HIBCH* | 3-hydroxyisobutyryl-CoA hydrolase | 0.68 | 0.028 |
| 9818_at | *NUPL1* | Nucleoporin like 1 | 0.85 | 0.028 |
| 151126_at | *ZNF385B* | Zinc finger protein 385B | 1.2 | 0.028 |
| 64759_at | *TNS3* | Tensin 3 | -0.77 | 0.028 |
| 29940_at | *DSE* | Dermatan sulfate epimerase | 0.79 | 0.029 |
| 4358_at | *MPV17* | MpV17 mitochondrial inner membrane protein | -0.86 | 0.029 |
| 768_at | *CA9* | Carbonic anhydrase IX | -1.48 | 0.029 |
| 7803_at | *PTP4A1* | Protein tyrosine phosphatase type IVA, member 1 | 0.4 | 0.029 |
| 6659_at | *SOX4* | SRY (sex determining region Y)-box 4 | 1.41 | 0.029 |
| 51299_at | *NRN1* | Neuritin 1 | 1.02 | 0.029 |
| 79971_at | *WLS* | Wntless homolog (Drosophila) | -0.71 | 0.029 |
| 51014_at | *TMED7* | Transmembrane emp24 protein transport domain containing 7 | 0.54 | 0.03 |
| 347862_at | *PDDC1* | Parkinson disease 7 domain containing 1 | -0.46 | 0.03 |
| 51310_at | *SLC22A17* | Solute carrier family 22, member 17 | -0.67 | 0.031 |
| 1891_at | *ECH1* | Enoyl CoA hydratase 1, peroxisomal | -0.4 | 0.031 |
| 7277_at | *TUBA4A* | Tubulin, alpha 4a | 0.79 | 0.031 |
| 80760_at | *ITIH5* | Inter-alpha-trypsin inhibitor heavy chain family, member 5 | -0.77 | 0.031 |
| 9358_at | *ITGBL1* | Integrin, beta-like 1 (with EGF-like repeat domains) | 1.17 | 0.032 |
| 84171_at | *LOXL4* | Lysyl oxidase-like 4 | -1.83 | 0.032 |
| 6662_at | *SOX9* | SRY (sex determining region Y)-box 9 | -0.38 | 0.032 |
| 9935_at | *MAFB* | V-maf musculoaponeurotic fibrosarcoma oncogene homolog B (avian) | 1.39 | 0.032 |
| 7341_at | *SUMO1* | SMT3 suppressor of mif two 3 homolog 1 (S. cerevisiae) | 0.64 | 0.032 |
| 5298_at | *PI4KB* | Phosphatidylinositol 4-kinase, catalytic, beta | -0.36 | 0.032 |
| 11221_at | *DUSP10* | Dual specificity phosphatase 10 | 0.84 | 0.032 |
| 3433_at | *IFIT2* | Interferon-induced protein with tetratricopeptide repeats 2 | 1.89 | 0.032 |
| 23764_at | *MAFF* | V-maf musculoaponeurotic fibrosarcoma oncogene homolog F (avian) | 0.47 | 0.033 |
| 10540_at | *DCTN2* | dynactin 2 (p50) | -0.42 | 0.033 |
| 8738_at | *CRADD* | CASP2 and RIPK1 domain containing adaptor with death domain | -0.64 | 0.033 |
| 26205_at | *GMEB2* | Glucocorticoid modulatory element binding protein 2 | -0.45 | 0.033 |
| 109_at | *ADCY3* | Adenylate cyclase 3 | -0.65 | 0.034 |
| 5265_at | *SERPINA1* | Serpin peptidase inhibitor, clade A (alpha-1 antiproteinase, antitrypsin), member 1 | -0.92 | 0.034 |
| 57600_at | *FNIP2* | Folliculin interacting protein 2 | 0.49 | 0.035 |
| 2810_at | *SFN* | Stratifin | -1.39 | 0.035 |
| 1203_at | *CLN5* | Ceroid-lipofuscinosis, neuronal 5 | 0.42 | 0.035 |
| 55041_at | *PLEKHB2* | Pleckstrin homology domain containing, family B (evectins) member 2 | 0.64 | 0.035 |
| 3216_at | *HOXB6* | Homeobox B6 | -0.61 | 0.035 |
| 522_at | *ATP5J* | ATP synthase, H+ transporting, mitochondrial Fo complex, subunit F6 | 0.57 | 0.035 |
| 10920_at | *COPS8* | COP9 constitutive photomorphogenic homolog subunit 8 (Arabidopsis) | 0.33 | 0.035 |
| 11186_at | *RASSF1* | Ras association (RalGDS/AF-6) domain family member 1 | -0.66 | 0.035 |
| 1030_at | *CDKN2B* | Cyclin-dependent kinase inhibitor 2B (p15, inhibits CDK4) | 0.67 | 0.035 |
| 677_at | *ZFP36L1* | Zinc finger protein 36, C3H type-like 1 | 1.19 | 0.035 |
| 390_at | *RND3* | Rho family GTPase 3 | 0.71 | 0.035 |
| 55854_at | *ZC3H15* | Zinc finger CCCH-type containing 15 | 0.25 | 0.035 |
| 23534_at | *TNPO3* | Transportin 3 | -0.4 | 0.035 |
| 4350_at | *MPG* | N-methylpurine-DNA glycosylase | -0.59 | 0.036 |
| 51191_at | *HERC5* | HECT and RLD domain containing E3 ubiquitin protein ligase 5 | 1.01 | 0.036 |
| 7205_at | *TRIP6* | Thyroid hormone receptor interactor 6 | -0.44 | 0.037 |
| 84190_at | *METTL25* | Methyltransferase like 25 | -0.59 | 0.037 |
| 10959_at | *TMED2* | Transmembrane emp24 domain trafficking protein 2 | 0.6 | 0.037 |
| 22822_at | *PHLDA1* | Pleckstrin homology-like domain, family A, member 1 | 0.77 | 0.039 |
| 3073_at | *HEXA* | Hexosaminidase A (alpha polypeptide) | -0.62 | 0.039 |
| 10051_at | *SMC4* | Structural maintenance of chromosomes 4 | 0.62 | 0.039 |
| 146198_at | *ZFP90* | Zinc finger protein 90 homolog (mouse) | -0.48 | 0.04 |
| 79003_at | *MIS12* | MIS12, MIND kinetochore complex component, homolog (S. pombe) | 0.65 | 0.04 |
| 6541_at | *SLC7A1* | Solute carrier family 7 (cationic amino acid transporter, y+ system), member 1 | -0.39 | 0.04 |
| 5277_at | *PIGA* | Phosphatidylinositol glycan anchor biosynthesis, class A | 0.54 | 0.041 |
| 85463_at | *ZC3H12C* | Zinc finger CCCH-type containing 12C | 0.89 | 0.041 |
| 8780_at | *RIOK3* | RIO kinase 3 (yeast) | 0.28 | 0.041 |
| 10914_at | *PAPOLA* | Poly(A) polymerase alpha | 0.38 | 0.042 |
| 727_at | *C5* | Complement component 5 | -0.7 | 0.042 |
| 81618_at | *ITM2C* | Integral membrane protein 2C | -0.71 | 0.042 |
| 55332_at | *DRAM1* | DNA-damage regulated autophagy modulator 1 | 0.49 | 0.042 |
| 83737_at | *ITCH* | Itchy E3 ubiquitin protein ligase | 0.38 | 0.042 |
| 80194_at | *TMEM134* | Transmembrane protein 134 | -0.64 | 0.042 |
| 1856_at | *DVL2* | Dishevelled, dsh homolog 2 (Drosophila) | -0.32 | 0.042 |
| 10529_at | *NEBL* | Nebulette | -0.47 | 0.043 |
| 84061_at | *MAGT1* | Magnesium transporter 1 | 0.37 | 0.043 |
| 285362_at | *SUMF1* | Sulfatase modifying factor 1 | -0.79 | 0.044 |
| 26053_at | *AUTS2* | Autism susceptibility candidate 2 | -0.46 | 0.044 |
| 57580_at | *PREX1* | Phosphatidylinositol-3,4,5-trisphosphate-dependent Rac exchange factor 1 | -0.61 | 0.044 |
| 79780_at | *CCDC82* | Coiled-coil domain containing 82 | 0.45 | 0.044 |
| 2297_at | *FOXD1* | Forkhead box D1 | 0.55 | 0.044 |
| 8639_at | *AOC3* | Amine oxidase, copper containing 3 (vascular adhesion protein 1) | -0.86 | 0.044 |
| 168451_at | *THAP5* | THAP domain containing 5 | 0.63 | 0.044 |
| 154_at | *ADRB2* | Adrenoceptor beta 2, surface | -0.97 | 0.044 |
| 25816_at | *TNFAIP8* | Tmor necrosis factor, alpha-induced protein 8 | 1.34 | 0.044 |
| 7469_at | *WHSC2* | Wolf-Hirschhorn syndrome candidate 2 | -0.29 | 0.044 |
| 163732_at | *CITED4* | Cbp/p300-interacting transactivator, with Glu/Asp-rich carboxy-terminal domain, 4 | -0.64 | 0.045 |
| 4863_at | *NPAT* | Nuclear protein, ataxia-telangiectasia locus | 0.46 | 0.046 |
| 29080_at | *CCDC59* | Coiled-coil domain containing 59 | 0.43 | 0.046 |
| 55638_at | *SYBU* | Syntabulin (syntaxin-interacting) | -0.72 | 0.046 |
| 79191_at | *IRX3* | Iroquois homeobox 3 | 1.45 | 0.046 |
| 22943_at | *DKK1* | Dickkopf 1 homolog (Xenopus laevis) | -1.25 | 0.046 |
| 84302_at | *TMEM246* | Transmembrane protein 246 | -0.67 | 0.046 |
| 4330_at | *MN1* | Meningioma (disrupted in balanced translocation) 1 | -1.01 | 0.047 |
| 285381_at | *DPH3* | DPH3, KTI11 homolog (S. cerevisiae) | 0.57 | 0.048 |
| 79600_at | *TCTN1* | Tectonic family member 1 | -0.54 | 0.048 |
| 4129_at | *MAOB* | Monoamine oxidase B | -0.92 | 0.048 |
| 4817_at | *NIT1* | Nitrilase 1 | -0.39 | 0.049 |
| 23531_at | *MMD* | Monocyte to macrophage differentiation-associated | 0.96 | 0.049 |
| 100506311_at | *HOTAIRM1* | HOXA transcript antisense RNA, myeloid-specific 1 | 1.25 | 0.05 |
| 51430_at | *SUCO* | SUN domain containing ossification factor | 0.44 | 0.05 |
| 54102_at | *CLIC6* | Chloride intracellular channel 6 | -0.52 | 0.05 |
